# Safety and effectiveness of diazepam nasal spray in patients with Rett syndrome and seizure clusters: *post hoc* analyses from a long-term safety study and survey of severity and burden

**DOI:** 10.3389/fneur.2026.1702423

**Published:** 2026-02-23

**Authors:** Daniel Tarquinio, James W. Wheless, Eric B. Segal, Adrian L. Rabinowicz, Enrique Carrazana

**Affiliations:** 1 Center for Rare Neurological Diseases, Norcross, GA, United States; 2 Le Bonheur Children’s Hospital, University of Tennessee Health Science Center, Memphis, TN, United States; 3 Hackensack University Medical Center and Northeast Regional Epilepsy Group and Hackensack Meridian School of Medicine, Hackensack, NJ, United States; 4 Neurelis, Inc., San Diego, CA, United States; 5 Center for Molecular Biology and Biotechnology, Charles E. Schmidt College of Science, Florida Atlantic University, Jupiter, FL, United States; 6 John A. Burns School of Medicine, University of Hawaii, Honolulu, HI, United States

**Keywords:** caregiver, developmental epileptic encephalopathy, natural variability, questionnaire, rescue therapy, SEIVAL, seizure clusters

## Abstract

**Introduction:**

Rett syndrome is a neurodevelopmental disorder associated with epilepsy that influences motor/communication skills, behavior, and other systems. Clinical experiences for these patients are not well described.

**Methods:**

This *post hoc* analysis consists of a patient subgroup with Rett syndrome enrolled in an open-label, single-arm, safety study. Age- and weight-based doses of diazepam nasal spray (5–20 mg) were administered to patients (aged 6–65 years) for seizure clusters. Treatment-emergent adverse events (TEAEs) were recorded, and days between seizure clusters (SEIzure interVAL [SEIVAL]) from Period 1 (P1; days 1–90) to Period 4 (P4; days 271–360) were calculated. Caregivers completed surveys describing experiences with diazepam nasal spray and Rett syndrome.

**Results:**

Rates of TEAEs (87.5%), serious TEAEs (12.5%), and treatment-related TEAEs (18.8%) in patients with Rett syndrome (*n* = 16) who received diazepam nasal spray were comparable to the overall pediatric safety population (87.2, 35.9, and 14.1%, respectively; *n* = 78), as was use of second doses (proxy for effectiveness) (18.4 and 11.4%). Increase in SEIVAL was also similar (P1 = 7.6, P4 = 25.0 days and P1 = 13.0, P4 = 25.9 days). Most (*n* = 12; 75.0%) remained in the study ≥12 months. All caregivers felt diazepam nasal spray was very/extremely easy to use; 63.6% were able to return to normal activities within an hour of administration. Caregiver perceptions of clinical severity and caregiver affect were stable from baseline to final visits; scores for individuals using diazepam nasal spray were numerically higher.

**Conclusion:**

Diazepam nasal spray has safety and effectiveness profiles consistent with the full study population and was easy to use.

**Clinical trial registration:**

ClinicalTrials.gov, NCT02721069.

## 1 Introduction

Rett syndrome is a neurodevelopmental disorder that involves a loss of motor and cognitive function and communication skills, as well as abnormal function of other physiologic systems (e.g., cardiovascular and respiratory) (1–3). As it is an X-linked disorder, Rett syndrome occurs in females and, rarely, males, and is commonly characterized by a mutation in the *MECP2* gene, with early symptoms emerging around 6–18 months of age (2, 3). Females with Rett syndrome typically demonstrate age-appropriate psychomotor development prior to symptom onset, followed by a brief developmental plateau before a rapid, marked loss of communication and motor skills that eventually stabilizes over time (4).

Among the neurologic manifestations of Rett syndrome, epilepsy has been reported in approximately 55–94% of cases (5–8). A prospective, longitudinal study estimated a cumulative lifetime prevalence of epilepsy in patients with Rett syndrome that approached 90%, with drug-resistant epilepsy noted in 16% (8). Seizure frequency and severity have been associated with Rett-syndrome diagnosis, and many patients can experience periods of seizure remission and relapse over time (8). For these patients and their parents, seizures can present a substantial, additional challenge to normal daily activities and quality of life (9).

People with epilepsy, including those with Rett syndrome, may experience seizure clusters, which are sometimes defined as ≥2 seizures that occur within a 24-h period (10). Seizure clusters are highly dependent on the specific patient, and early rectal diazepam studies identified seizure clusters based on their generally being distinguishable from a patient’s typical seizure pattern and easily recognizable by caregivers (11). However, the clinical course for patients with Rett syndrome who experience seizure clusters is poorly understood. A long-term, open-label safety study of diazepam nasal spray examined the safety of diazepam nasal spray for the treatment of seizure clusters in patients aged 6–65 years with intractable epilepsy (12), which included a subset of patients with Rett syndrome (13). Diazepam nasal spray is approved by the US Food & Drug Administration for the acute treatment of intermittent, stereotypic episodes of frequent seizure activity (i.e., seizure clusters and acute repetitive seizures) that are distinct from a patient’s usual seizure pattern in patients with epilepsy aged ≥2 years (14). Here, we present outcomes from the patients with Rett syndrome who participated in the safety study of diazepam nasal spray, as well as characterize the nature of Rett syndrome in this cohort that experienced seizure clusters.

## 2 Materials and methods

### 2.1 Study design

This is a *post hoc* analysis of a phase 3, open-label, repeat-dose, single-arm, safety study of diazepam nasal spray that took place from April 2016 to July 2020 (ClinicalTrials.gov identifier: NCT02721069). Complete methodology and results of the full study have been published (12). An institutional review board approved the study protocol and documentation (protocol number, 20152864), and the study was conducted in accordance with the Declaration of Helsinki and Good Clinical Practice. Participants or parents/guardians provided written informed consent before participation. Diazepam nasal spray was administered for treatment of seizure clusters for 12 months; patients could remain on treatment after the 12-month study period (12). Caregivers completed 2 sets of surveys: One characterized their experience in the long-term safety study of diazepam nasal spray, and the other was a parallel survey that focused on the nature of Rett syndrome in this cohort with seizure clusters.

### 2.2 Patients

Briefly, the long-term safety study enrolled patients who had a clinical diagnosis of epilepsy and, in the opinion of the investigator, might need treatment with a benzodiazepine for control of seizures an average of once every other month (i.e., 6 times per year) despite a stable regimen of antiseizure medications (ASMs) (12). Inclusion criteria consisted of the following: male or female patients between the ages of 6 and 65 years; diagnosis of partial or generalized epilepsy with motor seizures or seizures with clear alteration of awareness; availability of a qualified caregiver or medical professional capable of administering the study medication; and no abnormal findings of clinical significance in the patient’s medical history or upon physical examination, electrocardiogram, or clinical laboratory results at screening. A history of status epilepticus, seasonal allergies/rhinitis, or use of concomitant benzodiazepines did not disqualify patients from the study (15). Patients were excluded if they had a history of a clinically significant condition that would jeopardize their safety, active major depression, or suicide attempt or ideation (12). Patients included in this *post hoc* analysis were identified by the investigators.

### 2.3 Procedures

In the long-term safety study, diazepam nasal spray was administered in doses of 5, 10, 15, or 20 mg, according to patient age and weight. A second dose could be administered 4–12 h after the first (12). Dosage and timing of second doses could be adjusted by the investigators as clinically warranted (16). Seizure timing and drug administration were recorded in patient diaries (12). Caregivers completed an in-study survey of their experience with diazepam nasal at a time point near the end of the long-term safety study (10). Survey items included comfort with use of diazepam nasal spray, return to their own normal activities following administration, and convenience of diazepam nasal spray compared with rectal diazepam. The full methodology and overall results of this survey have been published (17).

The parallel survey that described the nature of Rett syndrome was assessed separately at 2 time points, which did not necessarily coincide with those of the long-term safety study. This Rett syndrome–specific survey consisted of items in the categories (instruments) of global severity (Clinical Global Impressions scales for Severity [CGI-S] and Improvement, Rett Syndrome Behaviour Questionnaire [RSBQ]) and caregiver affect (Positive and Negative Affect Schedule scale [PANAS]). The CGI-S characterizes the global severity of impairment, ranging from 1 (normal, not at all impaired) to 7 (the most impaired); Clinical Global Impressions–Improvement assesses clinical improvement from baseline on a scale from 1 to 7, where 1 is very much improved and 7 is very much worse (18). The RSBQ is a behavioral assessment that consists of 45 items, with each item scored on a 3-point Likert scale from 0 (the proposed behavior does not describe the person) to 2 (the proposed behavior often describes the person); total score ranges from 0 to 90 (higher score is indicative of severity) (19). The PANAS consists of two 10-item mood scales that characterize positive and negative affect (e.g., excited, ashamed); item responses range from 1 (very slightly or not at all) to 5 (extremely), with a total score for each scale ranging from 10 to 50 (20). High scores of positive affect indicate an enthusiastic, alert mood, while low scores indicate sadness and lethargy. In contrast, high scores of negative affect indicate various mood states, such as contempt and fear, whereas low scores indicate calmness (20).

### 2.4 Outcomes

The incidence and severity of treatment-emergent adverse events (TEAEs) were recorded, including those attributed to treatment (treatment-related TEAEs) (12). The number of second doses of diazepam nasal spray administered within 24 h of the first was used as a proxy for effectiveness (12), and the duration between seizure clusters (SEIzure interVAL [SEIVAL]) over time was examined (21). SEIVAL was assessed across 4 consecutive 90-day periods, and only patients who had ≥1 SEIVAL (i.e., the interval in days between 2 seizure clusters) for each period were included in the SEIVAL analysis. This consistent cohort was intended to address potential confounding inherent to assessment of a variable cohort over time (21). Responses to surveys of caregiver experience with diazepam nasal spray and the nature of Rett syndrome (global severity, caregiver affect) in patients with seizure clusters were tabulated. Additionally, items of the Rett syndrome survey were explored in the context of diazepam nasal spray usage. Data are expressed as raw scores, mean (SD), median (IQR), or patient counts (proportions). Descriptive statistics were used for this *post hoc* analysis, which was not powered to demonstrate statistical significance. Outcomes in the subgroup of patients with Rett syndrome were compared with those of the overall pediatric population in the larger safety study to assess the consistency of treatment effects.

## 3 Results

Of the 175 patients enrolled in the full long-term safety study (12), a subgroup of 20 had a diagnosis of Rett syndrome. A total of 163 patients received ≥1 dose of diazepam nasal spray and were included in the safety population, 78 were pediatric patients aged 6–17 years, and 16 of these had Rett syndrome (12, 22). This cohort of 16 patients with Rett syndrome was exclusively female, with ages ranging from 6 to 15 years (Table 1). Mean (SD) usage of diazepam nasal spray was 3.0 (2.2) doses per month in these patients. A total of 418 seizure clusters were treated in these patients. Duration of treatment exposure varied for patients with Rett syndrome, with 1 (6.3%) patient receiving treatment for <6 months, 3 (18.8%) receiving treatment for 6 to <12 months, and 12 (75.0%) patients receiving ≥12 months treatment.

**Table 1:** Patient characteristics.

Characteristic	Rett syndrome (*n* = 16)
Sex, female, *n* (%)	16 (100)
Race, *n* (%)	
White	15 (93.8)
Black or African American	1 (6.3)
Age, mean (SD), y	9.0 (2.8)
Weight, mean (SD), kg	28.5 (17.6)

### 3.1 Safety and effectiveness of diazepam nasal spray

Rates of TEAEs (87.5%), serious TEAEs (12.5%), and treatment-related TEAEs (18.8%) in patients with Rett syndrome who received diazepam nasal spray were generally similar to the overall pediatric safety population (87.2, 35.9, and 14.1%, respectively), although the rate of serious TEAEs was numerically lower (Table 2) (13, 22). No serious TEAEs or TEAEs leading to discontinuation were deemed related to treatment.

**Table 2:** Comparison of safety data.

Variable	Rett syndrome (*n* = 16)	Pediatric safety population (*n* = 78) (22)	Overall safety population (*N* = 163) (12)
Patients with TEAEs, *n* (%)	14 (87.5)	68 (87.2)	134 (82.2)
Treatment-related TEAEs	3 (18.8)	11 (14.1)	30 (18.4)
Patients with serious TEAEs, *n* (%)^*^	2 (12.5)	28 (35.9)	50 (30.7)
Required/prolonged hospitalization^*^	2 (12.5)	27 (34.6)	44 (27.0)
TEAEs leading to discontinuation^*^	0	0	1 (0.6)
Death^*^	0	0	1 (0.6)

* None deemed related to study treatment.

In this population with Rett syndrome, 18.4% of treated seizure clusters were treated with a second dose of diazepam nasal spray within 24 h of the first dose, which was comparable to the 11.4% rate in the overall pediatric safety population, indicating that a substantial proportion of seizure clusters were controlled with a single dose (13, 22). Of the 16 patients with Rett syndrome, 7 had SEIVAL data for all four 90-day periods. In this consistent cohort, mean (SD) SEIVAL numerically increased from 7.6 (2.2) days at Period 1–25.0 (38.4) days at Period 4 (*p* = 0.26); this trend was comparable to the change from 13.0 (11.9) to 25.9 (28.6) days observed in the consistent cohort from the pediatric subgroup of the full study (*n* = 32/76, aged 6–17 y; *p* = 0.02) (21).

### 3.2 Long-term safety study survey about diazepam nasal spray

Eleven of 16 caregivers (68.8%) completed the in-study survey on experience with diazepam nasal spray. Of the caregivers who responded, 11 (100%) felt that diazepam nasal spray was either very or extremely easy to use, and 90% (9/10) were either very or extremely comfortable using it in public. In caregivers who had experience with diazepam rectal gel, 100% (8/8) felt that rectal gel was not at all easy to use compared with diazepam nasal spray. Almost two-thirds of caregivers (7/11, 63.6%) were able to return to their own normal activities within an hour of administration, and 81.8% (9/11) were very comfortable doing activities outside the home with diazepam nasal spray available.

### 3.3 Rett syndrome–specific survey

Of the 20 caregivers of patients with Rett syndrome enrolled in the long-term safety study, 10 completed the parallel Rett syndrome–specific survey. Two of the 10 patients whose caregivers completed the Rett syndrome survey discontinued the long-term safety study prior to dosing and were not included in that study’s safety population. The median (IQR) duration between baseline and final visits for this survey was 46 (38.0) days. The median total score of Rett-specific behavior (RSBQ) decreased from 38.0 at baseline to 35.0 at final visit (numerically improved). Global severity of Rett syndrome and caregiver affect were largely unchanged at final visit (Table 3).

**Table 3:** Rett syndrome survey.

Category	Variable	Baseline visit	Final visit
Global severity	RSBQ total score	38.0 (37.0–41.0)	35.0 (31.0–40.0)
	Caregiver CGI-S^a^	5.0 (4.0–6.0)	5.0 (4.0–6.0)
	Caregiver CGI-I^b^	NA	4.0 (3.0–4.0)
Caregiver affect (PANAS)	Caregiver positive affect^c^	36.0 (27.0–43.0)	37.0 (34.0–38.0)
	Caregiver negative affect^c^	14.5 (11.0–21.0)	19.0 (12.0–24.0)

CGI-I, Clinical Global Impressions–Improvement; CGI-S, Clinical Global Impressions–Severity; NA, not applicable; PANAS, Positive and Negative Affect Schedule; RSBQ, Rett Syndrome Behaviour Questionnaire.

Data expressed as median (IQR) unless otherwise noted.

a 1–normal, not at all impaired; 2–borderline impaired; 3–mildly impaired; 4–moderately impaired; 5–markedly impaired; 6–severely impaired; 7–the most impaired.

b 1–very much improved; 2–much improved; 3–minimally improved; 4–no change; 5–minimally worse; 6–much worse; 7–very much worse.

c Scale: 10–50. High scores of positive affect indicate an enthusiastic, alert mood, while low scores indicate sadness and lethargy. High scores of negative affect indicate various mood states, such as contempt and fear, whereas low scores indicate calmness (16).

#### 3.3.1 Patients receiving diazepam between survey visits

**Figure 1:**
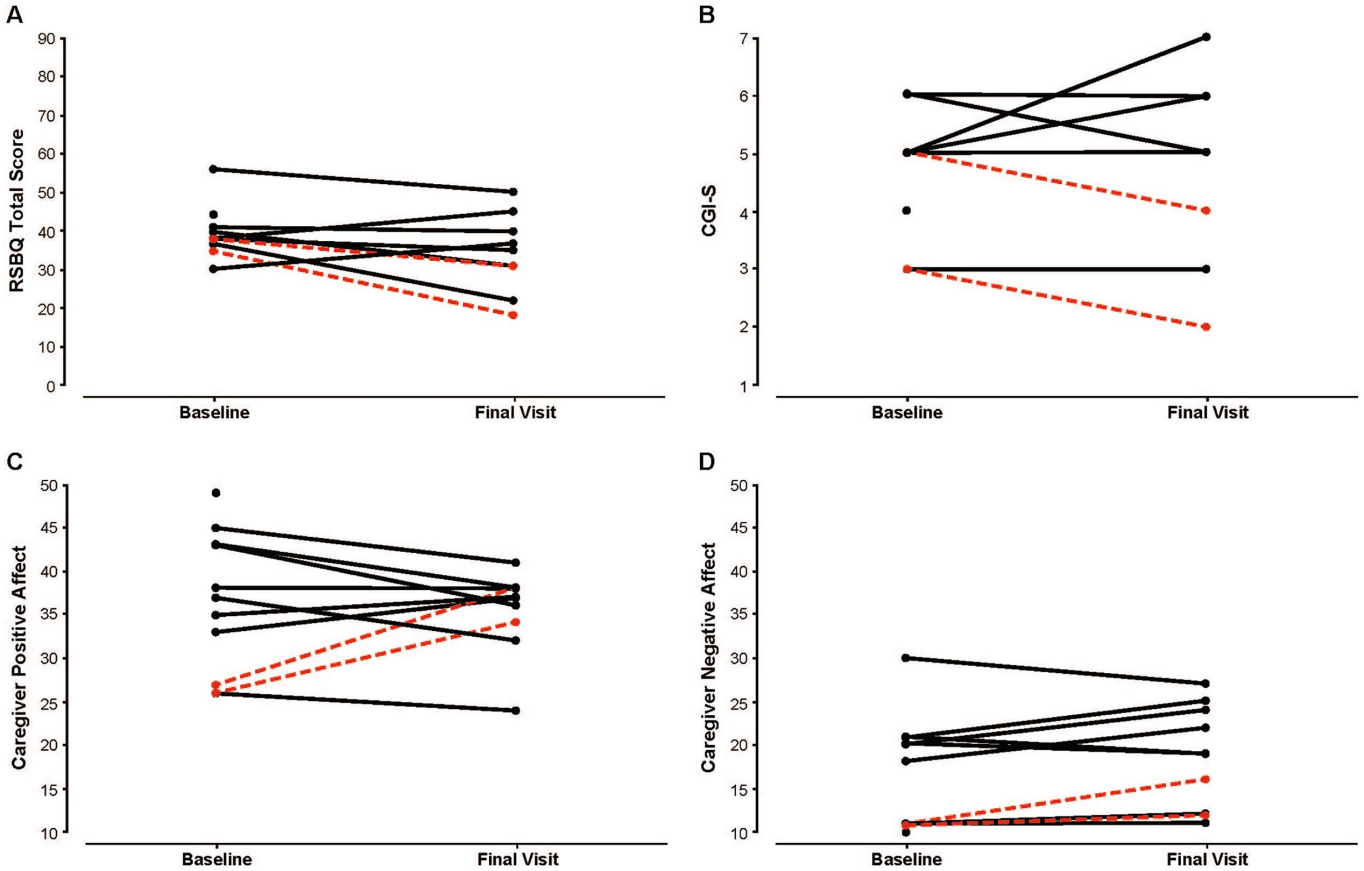
Global severity and caregiver affect at baseline and final visit. **(A)** Raw Rett-specific behavior (RSBQ) total scores. **(B)** Raw caregiver clinical severity (CGI-S) scores. ^a^ **(C)** Raw caregiver positive affect (PANAS) scores. **(D)** Raw caregiver negative affect (PANAS) scores. Scores in red indicate patients with recent frequent use of diazepam nasal spray. CGI-S, Clinical Global Impressions–Severity; PANAS, Positive and Negative Affect Schedule; RSBQ, Rett Syndrome Behaviour Questionnaire (higher values signify greater severity). ^a^1–normal, not at all impaired; 2–borderline impaired; 3–mildly impaired; 4–moderately impaired; 5–markedly impaired; 6–severely impaired; 7–the most impaired.

Of these 10 patients, 6 (60.0%) had *MECP2* mutations that have been associated with a severe phenotype of Rett syndrome (Table 4) (23). Four of the 10 patients (40.0%) received ≥1 dose of diazepam nasal spray between baseline and final visits, with a mean (SD) of 4.5 (3.9) doses per patient. Of these 4 patients, 2 had a history of recent frequent use prior to their final visit, with ≥3 doses within 30 days of their final visit. A reduction in Rett-specific behavior (RSBQ) score was associated with recent frequent use of diazepam nasal spray (Figure 1A), with the largest reduction (from 35 to 18) noted in the patient with greatest utilization of treatment between baseline and final visits (10 doses; dosed on the 2 days leading up to the final visit; mild Rett syndrome). The other patient with recent frequent use had an RSBQ reduction of 7 points. Modest reductions (directionally lower severity) in CGI-S scores were recorded for the 2 patients with recent frequent use of diazepam nasal spray (Figure 1B), from 5 and 3 at baseline to 4 and 2 at final visit. The largest increase in caregiver positive affect of the entire cohort occurred in the 2 caregivers of patients with recent frequent use of diazepam nasal spray (increased by 8 and 11 points) (Figure 1C). Caregivers of the 2 patients with recent frequent use of diazepam nasal spray had small increases in negative affect at the final visit (increased by 1 and 5 points; baseline score for both was 11 [lowest possible score is 10]) (Figure 1D).

**Table 4:** *MECP2*–mutation characteristics^a^.

Patient	*MECP2* mutation	Age, y	Severity	Diazepam
1	C1155_1172DEL18 CCTGCCCCCACCTCCACC	10	Mild	Not treated
2	R168X	6	Severe	Treated after surveys
3	R255X	7	Severe	Treated after surveys
4	753delC	8	Severe	Not treated
5	Deletion exons 3 and 4	12	Severe	Treated after surveys
6	P152R	8	Mild	Treated after surveys
7	R255X	8	Severe	Recent high use during surveys
8	1159_*6068del6371	15	Mild	Recent high use during surveys
9	c.806delG	7	Severe	Treated during surveys
10	IVS3-2A > g	9	Mild	Treated during surveys

a Patient cohort that completed the Rett syndrome–specific survey.

## 4 Discussion

Here, we presented results from a subgroup of patients with Rett syndrome who participated in the long-term safety study of diazepam nasal spray. The safety and effectiveness of diazepam spray in patients with Rett syndrome were comparable to results obtained in the overall pediatric safety population, as was the caregiver experience with diazepam nasal spray. In the Rett syndrome–specific survey, perceptions of clinical severity of Rett syndrome and caregiver mood in the overall group were relatively stable between baseline and final visits; however, there was intra-patient variability across time. Treatment with diazepam nasal spray accompanied some favorable changes in caregiver assessments, but the numbers of treated and untreated patients were very small.

While the burden of seizures in Rett syndrome has been described (24), there are few data on outcomes associated with acute management (immediate-use seizure medication) of seizure clusters in patients with Rett syndrome. In this subgroup of patients with Rett syndrome, the safety of diazepam nasal spray was comparable to the results obtained in the overall pediatric safety population, with patients with Rett syndrome experiencing a lower rate of serious TEAEs (12.5% vs. 35.9%) and similar rates of overall (87.5% vs. 87.2%) and treatment-related TEAEs (18.8% vs. 14.1%) (13, 22). The cohort of patients with Rett syndrome was considerably smaller than the overall safety study population, however, so these observations should be interpreted with caution. The effectiveness of diazepam nasal spray, using the proportion of patients who received a second dose within 24 h as a proxy measure, was comparable between the cohort of patients with Rett syndrome and the overall pediatric safety population (18.4% vs. 11.4%), as well as in the context of other studies of seizure-cluster treatment (13, 22, 25). In the increase in SEIVAL, which was noted in a *post hoc* analysis of the overall results (21), was also detected in the Rett syndrome cohort, albeit not statistically different in the small set of patients in this cohort who had SEIVAL data throughout the study. Taken together, these results support favorable safety and effectiveness profiles of diazepam nasal spray in patients with Rett syndrome, similar to that described in the overall pediatric safety population (aged 6–17 years) (13, 22).

For caregivers of patients with Rett syndrome, seizures can negatively influence quality of life (9). In the survey of caregivers, all respondents felt that diazepam nasal spray was easy to use, especially compared with diazepam rectal gel, which most had used in the past. Importantly, treatment with diazepam nasal spray was associated with a rapid return to their own normal activities (<1 h from administration) for almost two-thirds of caregivers, allowing caregivers (and patients) the opportunity to move forward with their day. The large proportion of caregivers (81.8%) who were comfortable with engaging in activities outside of the home with diazepam nasal spray on hand was suggestive that this type of treatment may alleviate some of the emotions/barriers (e.g., stressed, helpless) associated with seizure clusters (10).

The median clinical severity (CGI-S) of this cohort of patients with Rett syndrome was “markedly impaired” at baseline, and the median RSBQ total score at baseline (38.0) was consistent with the severity of Rett syndrome in this cohort (26, 27). Caregiver mood for this patient cohort was relatively calm, balanced between states of high energy/pleasurable engagement and lethargy. For caregiver affect (PANAS), caregiver responses to words that describe feelings and emotions associated with a positive affect (e.g., interested, alert, proud) (20) generally averaged in the “moderate” range (score of 3) at baseline and final visits. Responses to words that describe emotions associated with a negative affect (e.g., guilty, scared, nervous) tended to be at the lower end of the scale at baseline and final visits (median negative affect score, 14.5 and 19.0, respectively), indicative of a mostly peaceful mood (20).

Potential effects of diazepam nasal spray on global severity and caregiver affect are difficult to determine based on the small number of patients who were treated between baseline and final visits. However, we found that favorable changes in some items associated with clinical severity and caregiver affect were more common in patients who frequently used diazepam nasal spray. The patient with the greatest use of diazepam nasal spray (10 doses between baseline and final visits; 7 doses within 1 month of their final visit) had the largest reduction in Rett-specific behavior (RSBQ) total score (17 points; toward less severity) and increase in caregiver positive affect score (11 points; toward enthusiastic, alert mood) from baseline to final visit. The other patient who had 3 doses within 1 month of their final visit had the fourth-largest reduction in RSBQ total score (7 points; 7 patients had reductions) and second-largest increase in caregiver positive affect score (8 points). Of the 3 patients overall who were rated by their caregivers to have reduced clinical severity (CGI-S) at final visit, 2 were recent frequent users of diazepam nasal spray, although the reduction in CGI-S was by a single point for each patient.

### 4.1 Strengths and limitations

This study examined outcomes associated with diazepam nasal spray, an immediate-use seizure medication, in a group of patients (with Rett syndrome and intractable epilepsy, including seizure clusters) for which little is known. Limitations of this study include the small number of patients and lack of strict experimental control (e.g., use of treatment not randomized). In addition, of 20 total caregivers of patients with Rett syndrome, only 10 completed the Rett syndrome-specific survey. As a result, findings may not reflect experiences of all caregivers. Another potential limitation is that MECP2 testing was not required prior to enrollment; however, it was known for most patients in the study. Minimal clinically important differences in measures of global severity and caregiver affect are not well defined or have not been described. To our knowledge, this is the first time the PANAS scale has been used in Rett syndrome as an assessment of emotional affect; however, there are no similar Rett-specific scales to measure affect in caregivers. Finally, the degree of natural variability in function of patients with Rett syndrome (19) may have affected our findings.

### 4.2 Conclusion

The safety and effectiveness, assessed by use of second doses of diazepam nasal spray, is consistent with the overall pediatric safety population of the long-term safety study, illustrating the value of this immediate-use seizure medication in patients with Rett syndrome. The use of diazepam nasal spray was associated with a return to, or confidence to engage in, normal activities. Results from a Rett syndrome–specific survey of caregivers for patients enrolled in the long-term safety study also provide new insights into variability of Rett syndrome over time.

## Data Availability

The original contributions presented in the study are included in the article, further inquiries can be directed to the corresponding author.
